# Edible Flowers as Bioactive Food Ingredients with Antidiabetic Potential: A Study on *Paeonia officinalis* L., *Forsythia* × *intermedia*, *Gomphrena globosa* L., and *Clitoria ternatea* L.

**DOI:** 10.3390/plants14162603

**Published:** 2025-08-21

**Authors:** Maciej Książkiewicz, Michalina Karczewska, Filip Nawrot, Karolina Grabowska, Marcin Szymański, Judyta Cielecka-Piontek, Elżbieta Studzińska-Sroka

**Affiliations:** 1 The Student Scientific Society of Poznan University of Medical Sciences, Section “Pharmacognosy”, Poznan University of Medical Sciences, 3 Rokietnicka Str., 60-806 Poznan, Poland; ksioze.m@gmail.com (M.K.); michalina.karczewska0112@gmail.com (M.K.); filipnawrot9@gmail.com (F.N.); 2 Chair of Pharmacognosy, Jagiellonian University Medical College, 9 Medyczna Str., 30-688 Cracow, Poland; karolina1.grabowska@uj.edu.pl; 3 Center for Advanced Technologies, Adam Mickiewicz University in Poznań, 10 Uniwersytetu Poznańskiego Str., 61-614 Poznan, Poland; marcin.szymanski@amu.edu.pl; 4 Department of Pharmacognosy and Biomaterials, Poznan University of Medical Sciences, 3 Rokietnicka Str., 60-806 Poznan, Poland; jpiontek@ump.edu.pl

**Keywords:** plants extracts, polyphenols, oxidative stress, anti-inflammatory activity, metabolic disorders

## Abstract

Type 2 diabetes is a serious public health problem in the 21st century. To find new substances supporting diabetes therapy, researchers are increasingly paying attention to the biological potential of edible flowers. This study assessed the antidiabetic potential of ethanol, 50% ethanol, and water extracts from *Paeonia officinalis* L., *Forsythia* × *intermedia*, *Gomphrena globosa* L., and *Clitoria ternatea* L. flowers. Extracts were tested for antioxidant activity (DPPH, ABTS, FRAP, CUPRAC, and Fe^2+^ chelation), enzyme inhibition (α-glucosidase, α-amylase, hyaluronidase, and cholinesterases), and anti-inflammatory effects (NO inhibition in LPS-stimulated RAW264.7 macrophages). Phytochemical composition was also analysed. Extracts of *P. officinalis* stood out with the highest total phenolic content (50% ethanol extract of *P. officinalis* 178.49 mg GAE/g) and total flavonoid content (aqueous extracts of *P. officinalis* 4.27 mg QE/g), high gallic acid level, and the effective inhibition of α-glucosidase and α-amylase (α-glucosidase inhibition 98–99% for all *P. officinalis* extracts, and α-amylase inhibition ~ 100% for ethanolic extract). Strong hyaluronidase (76.9–95.5%) and cholinesterase inhibition was also observed. *F.* × *intermedia* extracts were rich in rutin and chlorogenic acid and showed potent inhibitory effects on α-glucosidase (50% ethanol extract 91.59%), α-amylase (aqueous extract 89.35%), and hyaluronidase (aqueous extract 73.8%). Ethanol extracts of *G. globosa* exhibited a high α-amylase inhibition (93–95%). Although *C. ternatea* showed moderate antioxidant activity, it showed an apparent anti-inflammatory effect, effectively reducing NO production in activated macrophages for 50% ethanol extract. In summary, *P. officinalis* and *F.* × *intermedia* flowers are promising sources of extracts with antioxidant, antidiabetic, and anti-inflammatory effects supporting their use in further research on type 2 diabetes therapy.

## 1. Introduction

Type 2 diabetes mellitus (T2DM) is one of the most significant public health challenges worldwide, affecting approximately 1 in 11 adults [1]. Current estimates indicate that the number of people with diabetes, currently around 445 million, is expected to nearly double by 2050 [2]. The main risk factors contributing to T2DM development include obesity, a sedentary lifestyle, and diets high in processed foods, particularly those rich in sugars and refined grains [1]. T2DM is a complex metabolic disorder characterised by chronic hyperglycaemia, which is closely linked to oxidative stress and inflammation [3,4]. Oxidative damage has also been implicated in the foetal origins of adult metabolic diseases [5]. Inflammation plays a critical role in diabetes pathogenesis, with enzymes such as hyaluronidase contributing to vascular complications, including diabetic nephropathy [6,7,8]. Additionally, elevated acetylcholinesterase and butyrylcholinesterase plasma levels have been reported in T2DM patients, suggesting their involvement in disease progression [9,10]. Current diabetes management includes first-line therapy with metformin; however, its use is often limited by side effects and insufficient efficacy in some patients, necessitating the combination with newer agents such as DPP-4 inhibitors, GLP-1 receptor agonists, and SGLT-2 inhibitors [11]. Despite their therapeutic benefits, these drugs are also associated with adverse effects, including gastrointestinal discomfort and an increased infection risk [12,13,14]. Given these limitations, there is a growing interest in complementary strategies, including lifestyle changes and the use of functional foods—foods containing bioactive compounds, which are often plant polyphenols, with health benefits [15].

Polyphenols are a diverse group of plant secondary metabolites, including several biologically active subclasses such as flavonoids, phenolic acids, anthocyanins, and proanthocyanidins—all of which have been investigated for their potential antidiabetic effects. Flavonoids, the most abundant polyphenols in the human diet, can modulate glucose metabolism by inhibiting carbohydrate-digesting enzymes (e.g., α-glucosidase and α-amylase), enhancing insulin sensitivity, and improving pancreatic β-cell function [16]. Phenolic acids such as chlorogenic, caffeic, or gallic acid contribute to glycemic regulation by protecting β-cells from apoptosis, supporting their function, and exhibiting potent antioxidant activity [17,18]. Similarly, anthocyanins and proanthocyanidins, abundant in colourful fruits and flowers, have been shown to reduce insulin resistance, mitigate oxidative stress, and prevent diabetes-related complications such as nephropathy and retinopathy [19,20]. Both preclinical and clinical studies have demonstrated that polyphenol-rich plant extracts improve glycemic control and modulate inflammatory pathways implicated in diabetes pathogenesis [21,22]. Plant-based dietary components with hypoglycemic, antioxidant, and anti-inflammatory properties have shown promise in animal models for improving diabetes and its complications by modulating oxidative stress and inflammation [23]. In recent years, there has been a growing interest in plant ingredients and natural products supporting glycemic control in people with type 2 diabetes. An example is *Gymnema sylvestre* extracts in lozenges that reduce the perception of sweet tastes and may reduce sugar intake and body weight [24]. Research shows that β-glucan has found a use in functional foods, e.g., *Coprinus comatus* in candy bars, an ingredient supporting glycemic control [25]. EFSA-approved microalgae such as *Arthrospira* and *Chlorella* provide prebiotic polysaccharides that support gut health and may beneficially influence glucose metabolism [26], and inositol, naturally present in legumes and whole grains, among others, has also been shown to improve insulin sensitivity and fasting glucose in clinical trials [27]. Among edible flowers, many of which are rich in polyphenols, several have demonstrated promising antidiabetic effects in vitro and in vivo. For example, *Hibiscus sabdariffa* has shown efficacy in both preclinical and clinical settings [28]. Other edible flowers, such as acacia, daisy, chamomile, cornflower, lavender, marigold (*Tagetes erecta*), and pumpkin, have been reported to inhibit α-glucosidase and/or α-amylase in vitro, and also exhibit antioxidant, anti-inflammatory, and cholinesterase-inhibiting properties [29,30]. These multifaceted properties may synergistically contribute to their antidiabetic potential. It therefore seems reasonable to assume that edible flowers can serve as bioactive food ingredients for diabetes prevention. Including them in daily diets—as infusions, edible dish decorations, or in formulated products—may offer health benefits beyond basic nutrition, and provide valuable support for conventional therapies in chronic diseases, including diabetes, by alleviating inflammation, reducing oxidative stress, and improving glycemic control.

Many edible flowers also have traditional medicinal uses that suggest biological activity. For instance, *Clitoria ternatea* is used in Indonesia, India, and Cuba for various ailments, including fever, eye diseases, and liver problems [31]. *Gomphrena globosa* is traditionally employed in India, Bangladesh, and Latin America for hypertension, jaundice, and urinary issues [32]. In Chinese medicine, *Paeonia lactiflora* flowers are used for menstrual irregularities, depression, and skin health [33]. In contrast, *Forsythia* × *intermedia* is primarily known as an ornamental plant in Europe, with limited medicinal data [34]. The bioactivity of these plants is often attributed to their diverse phytochemical content, including polyphenols, terpenes, and essential oils. However, their culinary use and rich phytochemical content make them especially attractive for development as novel functional foods targeting metabolic disorders. Polyphenols, in particular, have demonstrated antidiabetic potential through the inhibition of carbohydrate-digesting enzymes such as α-glucosidase and α-amylase, as well as anti-inflammatory and antioxidant effects [35,36]. Despite traditional uses and preliminary data, a comprehensive evaluation of these four species regarding their antidiabetic potential and phytochemical profiles is lacking. Moreover, there is little information linking their chemical composition to bioactivity.

Therefore, this study aims to assess the biological activities—including antidiabetic, antioxidant, neuroprotective, and anti-inflammatory effects—of the extracts obtained from the selected edible flowers (*Paeonia officinalis* L., *Forsythia* × *intermedia*, *Gomphrena globosa* L., and *Clitoria ternatea* L.), alongside detailed phytochemical characterisation, to identify promising candidates for functional food development targeting T2DM prevention.

## 2. Results and Discussion

### 2.1. Phytochemical Studies of the Extracts

#### 2.1.1. Total Polyphenol Content (TPC) and Total Flavonoid Content (TFC)

**Figure 1:**
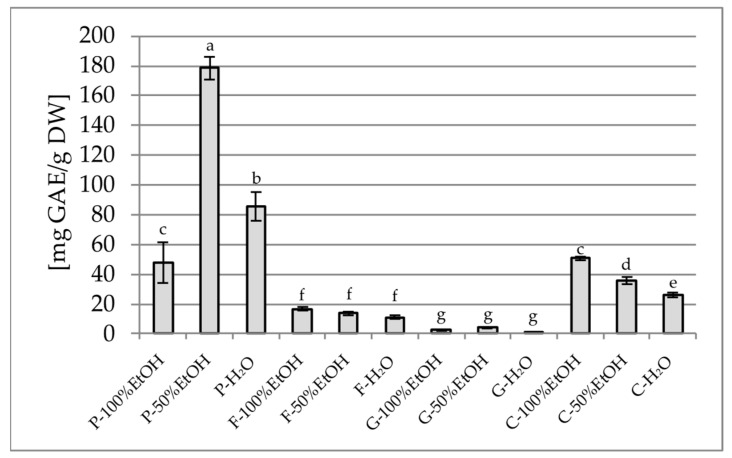
Total polyphenol content in the tested samples, expressed as mg gallic acid equivalent per gram of dry weight [mg GAE/g DW]. P—Paeonia officinalis, F—Forsythia × intermedia, G—Gomphrena globosa, C—Clitoria ternatea, H_2_O—aqueous extract, EtOH—ethanol extract. Values are presented as mean ± SD. Different letters above the bars indicate significant differences between groups (*p* < 0.05, one-way ANOVA followed by Newman–Keuls post hoc test).

**Figure 2:**
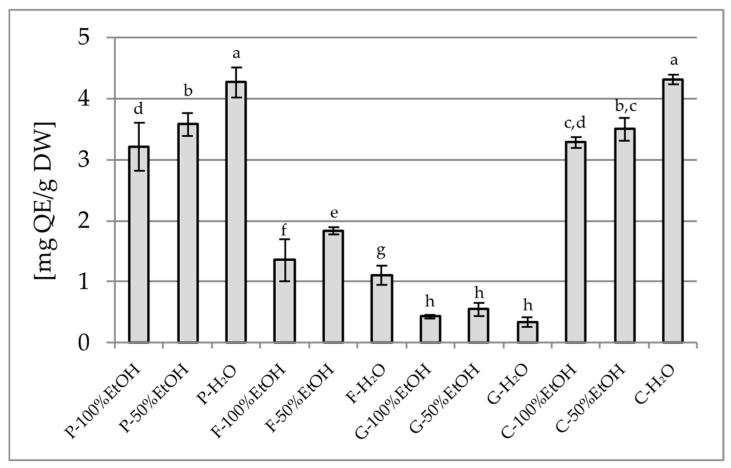
Total flavonoid content in the tested samples, expressed as mg quercetin equivalent per gram of dry weight [mg QE/g DW]. P—Paeonia officinalis, F—Forsythia × intermedia, G—Gomphrena globosa, C—Clitoria ternatea, H_2_O—aqueous extract, EtOH—ethanol extract. Values are presented as mean ± SD. Different letters above the bars indicate significant differences between groups (*p* < 0.05, one-way ANOVA followed by Newman–Keuls post hoc test).

The analysis of the total polyphenol content (TPC) and the total flavonoid content (TFC) revealed clear differences depending on both the plant species and the type of solvent used. The highest TPC was observed for the 50% ethanol extract of *P. officinalis* (178.49 mg GAE/g) (Figure 1), which significantly exceeded the values noted for the other extracts of this species and also the other plants included in the study. In contrast, the lowest TPC values were obtained for aqueous extracts of *G. globosa* (1.12 mg GAE/g). It is worth noting that aqueous extracts often showed a lower phenolic content than the 50% ethanol extracts, which may be due to the limited solubility of certain polyphenols in water. In terms of TFC, the highest values were found in aqueous extracts of *P. officinalis* (4.27 mg QE/g) and *C. ternatea* (4.31 mg QE/g) (Figure 2), while the lowest values were recorded in all extracts of *G. globosa* (0.34–0.56 mg QE/g). 

#### 2.1.2. Standardisation of the Extract Using High-Performance Liquid Chromatography (HPLC)

In the HPLC analysis, the content of four phenolic compounds was determined: chlorogenic acid, gallic acid, p-coumaric acid, ferulic acid, and quercetin-3-O-rutinoside (rutin) in ethanol, water, and 50% ethanol extracts from the flowers of *P. officinalis*, *F.* × *intermedia*, *G. globosa*, and *C. ternatea*. The obtained results (Table 1) showed significant qualitative and quantitative differences in the composition of phenolic compounds, depending on both the plant species and the type of solvent used. Moreover, it should be emphasised that the concentration of secondary metabolites varies depending on factors such as plant part, age, geographical origin, and extraction method [37,38].

The highest rutin content was found in the ethanol and 50% ethanol extracts from *F.* × *intermedia*, amounting to 2828.71 mg/100 g dry weight (DW) and 4657.71 mg/100 g DW, respectively. In the aqueous extract of *F.* × *intermedia*, rutin was present only in trace amounts (0.90 mg/100 g DW). The results show that the justification in TPC results was that water *F.* × *intermedia* extracts demonstrated a lower content of polyphenols and flavonoids. These results may indicate the presence of factors limiting its solubility or a specific form of this flavonoid in the plant material. Studies by various authors indicate that rutin is a flavonoid present in the flowers of different *Forsythia* species. Kicel et al. reported that rutin is one of the dominant compounds in *F.* × *intermedia*, with an even higher content noted in *F. suspensa* flowers. The rutin content in *F.* × *intermedia* flowers was similar to our analyses, amounting to 42.30 mg/g [39]. Moderate rutin concentrations were also detected in extracts from *P. officinalis* (167.65–239.59 mg/100 g DW) and the 50% ethanol extract from *C. ternatea* (160.35 mg/100 g DW). Although reports on rutin content in *P. officinalis* flowers are limited, Lee et al. indicated the presence of this compound in *Paeonia suffruticosa* flowers (334.26 µg/g) [40]. In contrast, Paz Gonçalves reported rutin in *C. ternatea* flowers [41]. Extracts from *G. globosa* contained rutin, but in small amounts. Chlorogenic acid was found in varying concentrations across the analysed flower extracts, with the highest content noted in the 50% ethanol extract from *F.* × *intermedia*, reaching 796.03 mg/100 g DW. A high amount was also detected in the ethanol extract of this species (319.79 mg/100 g DW), while in the aqueous extract, the content was negligible (0.31 mg/100 g DW). The presence of chlorogenic acid in the species *F.* × *intermedia* has been previously described [39]. Among the other species, *G. globosa* and *C. ternatea* extracts contained only trace amounts of chlorogenic acid (0.70–2.83 mg/100 g DW and 0.17–1.93 mg/100 g DW, respectively), which is consistent with previous reports [42,43]. In contrast, in *P. officinalis* flowers, chlorogenic acid was not detected in any of the tested extracts. Gallic acid was the dominant compound in *P. officinalis* flowers, with a content ranging from 390.27 ± 15.48 to 603.67 ± 10.10 mg/100 g DW, depending on the extract. The presence of gallic acid in the flowers of various *Paeonia* species has already been reported in the literature [44,45]. However, these studies are scarce. In the other examined flowers, gallic acid levels were trace or undetectable. According to the literature, ferulic acid presence was confirmed in extracts from *C. ternatea* [46] and in small amounts in *G. globosa* [47]. The ferulic acid content in *F.* × *intermedia* flowers was low and has not been previously reported in the literature. Meanwhile, p-coumaric acid was present in small amounts in all analysed flower species except for *P. officinalis*. The results clearly emphasise the significant influence of solvent type on the extraction efficiency of individual compounds. Generally, 50% ethanol extracts provided higher yields of rutin and ferulic acid than aqueous or pure ethanol extracts, which can be attributed to the optimal polarity of this solvent for a broad spectrum of phenolic compounds. The aqueous extract, however, was most effective in extracting gallic acid from *P. officinalis* flowers.

The results of our studies indicate that the compounds particularly abundant in the extracts studied are gallic acid in *P. officinalis* and rutin and chlorogenic acid in *F.* × *intermedia*. The scientific data available in the literature suggest that chlorogenic acid, gallic acid, and rutin have a multifaceted effect that may support treating type 2 diabetes through mechanisms related to improving insulin sensitivity and protecting pancreatic β cells. Chlorogenic acid has been shown to lower fasting blood glucose levels in clinical trials [48,49] and in in vivo studies [50]. In turn, gallic acid increases PPARγ expression. It activates the PI3K/Akt pathway, which leads to GLUT4 translocation to the plasma membrane, increased glucose uptake, and improved insulin sensitivity in a rat model of a high-fat diet and streptozotocin-induced diabetes [51,52,53]. Furthermore, gallic acid reduces fasting glycemia, improves hepatic glucose metabolism, and protects pancreatic β cells from oxidative stress through antioxidant mechanisms and the modulation of the Nrf2 pathway [54]. Rutin, on the other hand, has antihyperglycemic effects by stimulating insulin secretion, increasing glucose uptake, and protecting Langerhans β cells from degeneration [55,56,57]. At the same time, it reduces oxidative stress and the formation of advanced glycation end products (AGEs), which prevents the vascular damage typical of diabetic complications [58]. Furthermore, rutin ameliorates diabetic neuropathy by lowering plasma glucose and decreasing oxidative stress via the Nrf2 signalling pathway in rats [59].

#### 2.1.3. GC-MS Analysis

The GC-MS analysis of flowers from four plant species revealed the presence of several lipophilic compounds, including long-chain hydrocarbons (e.g., pentacosane, tetratetracontane, and hexatriacontane), fatty acid esters, alcohols, and steroid compounds. The dominant constituents included dihydrofuranone in *F.* × *intermedia* (79.67%), tetratetracontane in *G. globosa* (41.74%), and octadecane, 3-ethyl-5-(2-ethylbutyl)- in *C. ternatea* (49.10%) (Table 2). Data from the literature suggest that the identified substances show potential significance in the context of antidiabetic therapy. In the three analysed samples (*P. officinalis*, *F.*× *intermedia*, and *G. globosa*), oleic acid and its ester derivatives belonging to monounsaturated fatty acids were detected. These compounds have anti-inflammatory effects, increase tissue sensitivity to insulin, and improve the lipid profile, which has been confirmed in clinical and experimental studies [60]. In the *P. officinalis* and *G. globosa* samples, steroid derivatives (e.g., androstatriene or spirost-8-en-11-one) were also identified. The available evidence indicates that this class of compounds may protect pancreatic β-cells from oxidative stress and inhibit carbohydrate-digesting enzymes such as α-glucosidase, thereby potentially limiting postprandial blood glucose spikes [61]. Although many of the saturated alkanes detected in the studied edible flowers do not show direct pharmacological activity, their presence may affect the lipophilicity of the plant metabolite mixture. Increased lipophilicity may facilitate the transport of active substances in the body and stabilise their action. Studies on semifluorinated alkanes have shown that similar compounds act as effective carriers for pharmaceuticals, increasing their permeability and stability [62].

### 2.2. Studies of the Biological Activity of Extracts

Diabetes is a complex disease, involving not only glycemic disturbances but also dysfunctions of other organs, especially in elderly patients. Its prevention and control—particularly in the early stages—require a multidimensional approach. This includes lowering blood glucose levels and reducing oxidative stress, alleviating inflammation, and supporting cognitive functions. In light of these findings, and recognising the need for a multifaceted approach, our study evaluates the antidiabetic potential of aqueous, ethanol–aqueous, and ethanol extracts using a range of in vitro experimental methods.

#### 2.2.1. Antioxidant Activity Assay

Antioxidant activity plays a key role in maintaining human health, particularly in preventing and managing type 2 diabetes. Excessive reactive oxygen species (ROS) production and impaired antioxidant defences can damage pancreatic β-cells, contributing to insulin resistance and hyperglycemia [63]. Reducing oxidative stress is thus a justified complementary strategy. Epidemiological studies support this view, showing that diets rich in antioxidants (e.g., vitamin C) may reduce the risk of type 2 diabetes, particularly in adult women [64]. Moreover, reducing oxidative stress may help prevent diabetic complications by protecting the vascular endothelium, thus lowering the risk of retinopathy and nephropathy [65,66].

Our study demonstrated a high antioxidant potential in several edible flower extracts (Table 3). *P. officinalis* extracts (both water and ethanol-based) showed powerful antiradical activity in DPPH (90–98%) and ABTS (99–100%) tests. *F.* × *intermedia* extracts, especially the 50% ethanol variant, also showed considerable activity. In contrast, *C. ternatea* and *G. globosa* displayed lower antioxidant activity in both tests (DPPH < 30%, ABTS up to 54%). Interestingly, Fe^2+^ chelation was most effective in aqueous extracts, while the ethanolic ones showed no chelating ability at the tested concentrations. This suggests different mechanisms of antioxidant action: water extracts likely act via metal ion complexation (inhibiting the Fenton reaction), while ethanol extracts act through radical scavenging [62,67]. For a more comprehensive assessment of antioxidant potential, tests were also conducted to determine the ability of antioxidants to reduce metal ions (CUPRAC—Cu^2+^ ions to Cu^+^, FRAP—Fe^3+^ ions to Fe^2+^). These tests confirmed the strong antioxidant potential of *P. officinalis*, especially its ethanolic extracts (175.06 and 144.06 mg TE/g DW). Similar trends, with high activity observed for ethanol-based extracts, were observed for *F.* × *intermedia*, which is consistent with the results of our phytochemical studies indicating a lower chlorogenic acid and rutin content in the aqueous extract of *F.* × *intermedia* compared with alcoholic extracts. In contrast, both *C. ternatea* and *G. globosa* had consistently a low reducing capacity across all assays. These findings confirm that *P. officinalis* and *F.* × *intermedia* are promising sources of antioxidants and may aid in preventing oxidative stress-related diseases such as type 2 diabetes. Overall, the variation in the results between antioxidant assays highlights the importance of using a wide array of tests (DPPH, ABTS, Fe^2+^ chelation, CUPRAC, and FRAP) to assess the antioxidant activity [67] fully.

Reports in the literature support our findings. Different parts of *P. officinalis* (leaves, roots) have shown strong antioxidant properties [68], while *Paeonia ostii* flower extracts were effective in DPPH and ABTS tests [69]. *Forsythia koreana* inhibited superoxide radicals [70], and *Forsythia suspensa* activated antioxidant pathways (Nrf2/HO-1) in inflammatory models [71]. Our study’s low antioxidant activity of *C. ternatea* aligns with reports showing higher activity in aqueous than ethanol-based extracts. Similarly, though data on *G. globosa* is limited, some studies confirm the antioxidant properties of ethanolic macerates.

#### 2.2.2. Inhibition of Carbohydrate-Hydrolysing Enzymes

**Figure 3:**
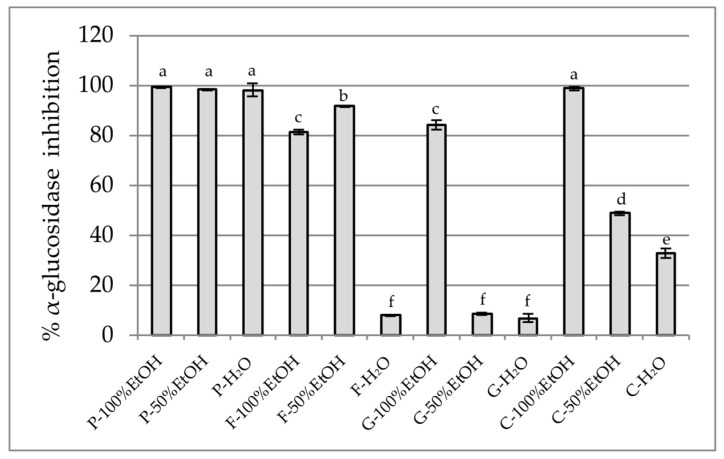
α-Glucosidase inhibitory potential of extracts (25 mg DW/mL). P—*Paeonia officinalis*, F—*Forsythia* × *intermedia*, G—*Gomphrena globosa*, C—*Clitoria ternatea*. H_2_O—aqueous extract, EtOH—ethanol extract. Values are presented as mean ± SD. Different letters above the bars indicate significant differences between groups (*p* < 0.05, one-way ANOVA followed by Newman–Keuls post hoc test).

The inhibition of digestive enzymes such as α-amylase and α-glucosidase is a well established strategy in controlling postprandial hyperglycemia in type 2 diabetes [72]. In this study, we evaluated the ability of selected edible flower extracts to inhibit these enzymes, using standard in vitro assays to assess their potential for modulating carbohydrate digestion. The first enzyme tested was α-glucosidase (Figure 3). Nearly complete inhibition was observed among the tested samples for all *P. officinalis* extracts (98–99%). Potent inhibition was also observed for the 100% ethanol extract of *C. ternatea* (98.7%), which is consistent with studies by Escher et al. that demonstrated the high α-glucosidase inhibition by lyophilised extracts of *C. ternatea* [73]. *F.* × *intermedia* and *G. globosa* exhibited potent inhibition only in selected extracts (91.59% in F-50% EtOH and 84.10% in G-100% EtOH, respectively). In comparison, aqueous extracts of *F.* × *intermedia* and *G. globosa* showed minimal activity (<10%), indicating that the active glucosidase inhibitors are primarily ethanol-soluble. Moreover, Tang et al. mentioned that significant inhibitory effects on α-glucosidase were observed with the phenolic compounds from *G. globosa* [74].

**Figure 4:**
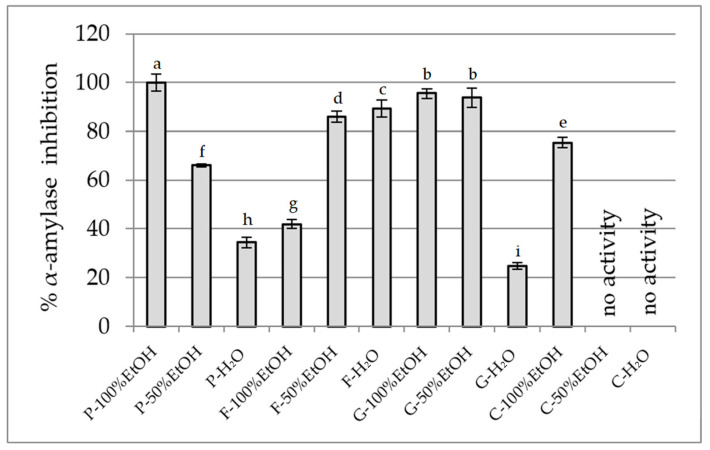
α-Amylase inhibitory potential of extracts (25 mg DW/mL). P—*Paeonia officinalis*, F—*Forsythia* × *intermedia*, G—*Gomphrena globosa*, C—*Clitoria ternatea*. H_2_O—aqueous extract, EtOH—ethanol extract. Values are presented as mean ± SD. Different letters above the bars indicate significant differences between groups (*p* < 0.05, one-way ANOVA followed by Newman–Keuls post hoc test).

In the case of α-amylase (Figure 4), the ethanolic extract of *P. officinalis* demonstrated the strongest activity (~100%), followed by ethanol-based extracts of *G. globosa* (93–95%). Interestingly, *F.* × *intermedia* exhibited high activity in the aqueous (89.35%) and 50% ethanol (85.90%) extracts, while the 100% ethanol extract was notably weaker (41.93%), suggesting that the active constituents may be more water-soluble. *C. ternatea* hydroalcoholic and aqueous extracts showed no measurable α-amylase inhibition.

Our findings complement other studies showing a high α-amylase-inhibitory effect for the root and leaf aqueous and methanol extracts, respectively, from *P. officinalis*, which inhibited α-amylase in a dose-dependent manner [68]. The results for *F.* × *intermedia*, including different species and parts of the plant, demonstrated that the leaves of *F. suspensa* showed high inhibition on α-glucosidase. Significantly, the inhibitory profiles differed substantially between the two enzymes, underscoring the need for dual-enzyme assays when evaluating the antidiabetic potential of plant extracts. Moreover, these results are particularly noteworthy as they suggest that *P. officinalis* and *F.* × *intermedia* flower extracts may exhibit stronger enzyme inhibitory activity than those of *C. ternatea* and *G. globosa*, which have documented but comparatively moderate effects [74,75,76,77,78], although the 100% ethanolic extract of *G. globosa* also shows considerable potential. Thus, our data point to the flower extracts of *P. officinalis* and *F.* × *intermedia* as promising candidates that are potentially superior to these previously studied species in terms of enzyme inhibition relevant to diabetes risk reduction.

#### 2.2.3. Anti-Inflammatory Activity Assay

Chronic inflammation plays a key role in the pathogenesis of type 2 diabetes and its complications [79]. Persistent low-grade inflammation contributes to insulin resistance, pancreatic β-cell dysfunction, and the progression of vascular complications. It has been proven that compounds of natural origin can alleviate ongoing inflammation [80]; hence, expanding knowledge in the biological activity of natural substances is an important research direction.

##### Hyaluronidase Inhibition Assay

One of the mechanisms recognised as an indicator of anti-inflammatory potential is the inhibition of hyaluronidase. High-molecular-weight hyaluronic acid stimulates anti-inflammatory responses. It tends to act as a physical barrier, preventing immune cell infiltration into tissues [81], while lower-molecular-weight molecules induce inflammation [82]. Moreover, hyaluronic acid also possesses antioxidative properties, and it is believed that the high-molecular-weight form of hyaluronic acid can protect against the effects of reactive oxygen species [83].

**Figure 5:**
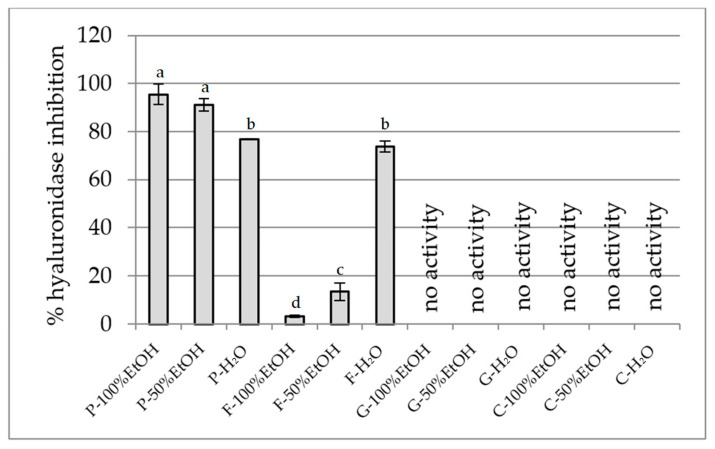
Hyaluronidase inhibitory potential of extracts (25 mg DW/mL). P—*Paeonia officinalis*, F—*Forsythia* × *intermedia*, G—*Gomphrena globosa*, C—*Clitoria ternatea*. H_2_O—aqueous extract, EtOH—ethanol extract. Values are presented as mean ± SD. Different letters above the bars indicate significant differences between groups (*p* < 0.05, one-way ANOVA followed by Newman–Keuls post hoc test).

Our findings highlight that among the edible flowers tested, *P. officinalis* and *F.* × *intermedia* possess a promising anti-hyaluronidase effect (Figure 5). *P. officinalis* extracts exhibited the strongest hyaluronidase inhibition, with values reaching 95.5% for the 100% ethanol extract, 91.1% for the 50% ethanol extract, and 76.9% for the aqueous extract. This high activity suggests the presence of potent anti-hyaluronidase compounds. In contrast, *F.* × *intermedia* showed a varied profile: low inhibition in the 100% ethanol extract (3.3%) and moderate to high in the 50% ethanol (13.4%) and aqueous extract (73.8%), indicating that water-soluble constituents are primarily responsible for its inhibitory effect. Notably, *G. globosa* and *C. ternatea* extracts demonstrated no hyaluronidase inhibition across all solvent extracts, suggesting limited potential in this anti-inflammatory pathway.

##### Determination of Anti-Inflammatory Potential in RAW 264.7 Model

**Figure 6:**
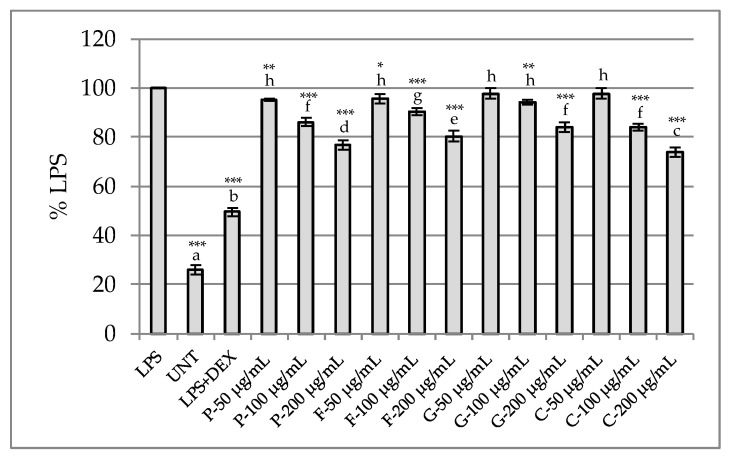
The effect of flower 50% ethanol extracts on nitric oxide release in LPS-stimulated RAW 264.7 macrophages. P—*Paeonia officinalis*, F—*Forsythia* × *intermedia*, G—*Gomphrena globosa*, C—*Clitoria ternatea*. LPS—lipopolysaccharide, UNT—untreated cells, DEX—dexamethasone (reference drug). Different letters above the bars indicate significant differences between groups (*p* < 0.05, one-way ANOVA followed by Newman–Keuls post hoc test). Asterisks indicate statistically significant differences vs. LPS-treated cells (* *p* < 0.05; ** 0.01 ≤ *p* < 0.001; *** *p* < 0.001, one-way ANOVA followed by Dunnett post hoc test).

The anti-inflammatory potential of the tested extracts was evaluated using the LPS-stimulated RAW267.4 murine macrophage model by measuring nitric oxide (NO) release. None of the tested samples were toxic to macrophages at the tested concentration range (up to 200 μg/mL), with cell viability comparable to untreated control cells. Thus, the anti-inflammatory assay was conducted across the entire concentration range. However, no anti-inflammatory activity was noted for the tested extracts at lower concentrations. Weak to moderate effects were noted at 100 and 200 μg/mL. The greatest inhibition of nitric oxide release was achieved with the *C. ternatea* and *P. officinalis* samples (Figure 6). The observed effect of the inhibition of NO release by the two extracts was significantly higher than that of LPS-stimulated cells.

Importantly, our analyses demonstrated that flower extracts from *P. officinalis* and *C. ternatea* contained the highest levels of polyphenols, including flavonoids, among all tested species. While no data have yet described NO inhibition by *P. officinalis*, the acetate fraction of *C. ternatea* flower extracts significantly and dose-dependently suppressed NO production [84]. The observed anti-inflammatory effect may be due to gallic acid and rutin present in the extracts, compounds that have been reported in the literature as inhibitors of NO synthesis [85,86]. Although flowers of *F.* × *intermedia* have not been studied in the RAW 264.7 murine macrophage model, data from the literature suggest that the butanol fraction from *Forsythia koreana* flowers inhibits NO production [87]. In contrast, *G. globosa* flowers have not been studied in this model before. Notably, the observed anti-inflammatory activity did not correlate with the antioxidant potential of the tested extracts. While *P. officinalis* flower extracts exhibited strong antioxidant activity, those from *C. ternatea* showed considerably weaker antioxidant effects.

#### 2.2.4. Anti-Cholinesterase Activity Assay

Type 2 diabetes is associated with numerous complications, including the dysfunction of both the central and peripheral nervous systems. Epidemiological studies indicate the increased risk of developing dementia and Alzheimer’s disease in people with diabetes [88], which is associated, among others, with the deficiency of the neurotransmitter acetylcholine (ACh) in the brain. The elevated activity of the enzymes acetylcholinesterase (AChE) and butyrylcholinesterase (BChE), responsible for breaking down ACh, contributes to the deterioration of cholinergic transmission in the nervous system [89]. Therefore, the inhibition of AChE and BChE represents a promising therapeutic strategy to protect cognitive functions and support metabolic control in patients with diabetes.

**Figure 7:**
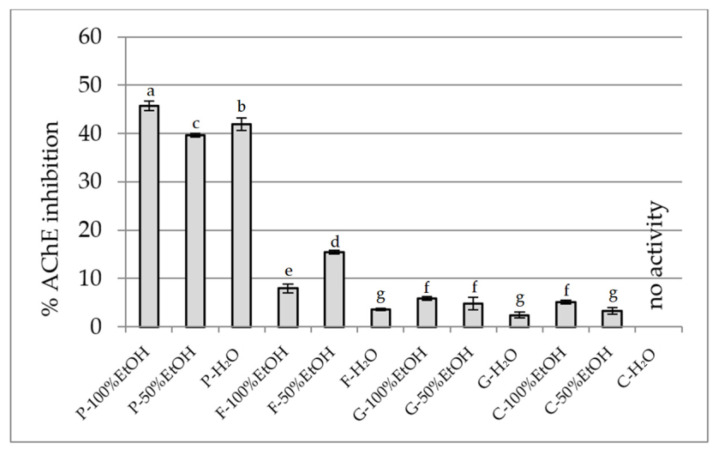
AChE inhibitory potential of extracts (25 mg DW/mL). P—*Paeonia officinalis*, F—*Forsythia* × *intermedia*, G—*Gomphrena globosa*, C—*Clitoria ternatea*. H_2_O—aqueous extract, EtOH—ethanol extract. Values are presented as mean ± SD. Different letters above the bars indicate significant differences between groups (*p* < 0.05, one-way ANOVA followed by Newman–Keuls post hoc test).

**Figure 8:**
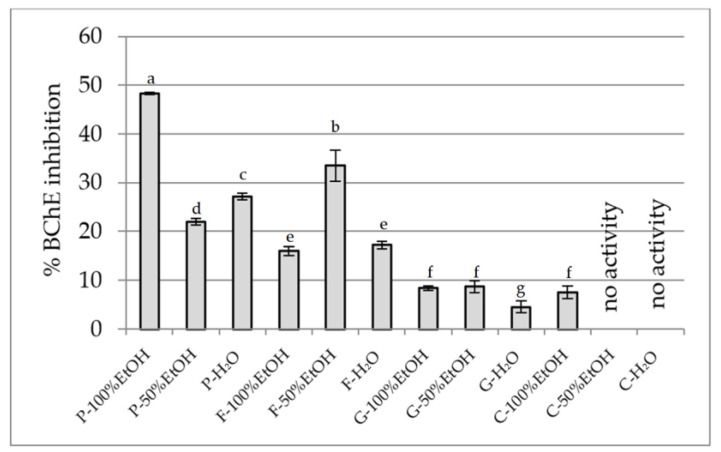
BChE inhibitory potential of extracts (25 mg DW/mL). P—*Paeonia officinalis*, F—*Forsythia* × *intermedia*, G—*Gomphrena globosa*, C—*Clitoria ternatea*. H_2_O—aqueous extract, EtOH—ethanol extract. Values are presented as mean ± SD. Different letters above the bars indicate significant differences between groups (*p* < 0.05, one-way ANOVA followed by Newman–Keuls post hoc test).

Based on this, the inhibitory potential of extracts from the flowers of *P. officinalis*, *Forsythia* × *intermedia*, *G. globosa,* and *C. ternatea* to inhibit AChE and BChE was assessed (Figure 7 and Figure 8, respectively). The highest activity against AChE was demonstrated by ethanol extracts of *P. officinalis* (~46%), as well as hydroalcoholic and aqueous extracts of this species (~40%). Considerably lower activity was observed for the extracts from *F.* × *intermedia* and *G. globosa* (usually <16%), while *C. ternatea* showed very weak activity (3–5%), with no effect in the aqueous extract. Similar inhibition patterns were found for BChE. The strongest activity was demonstrated by the ethanol extract from *P. officinalis* (48.3%), while the other extracts of this species inhibited the enzyme within a range of 22–27%. Moderate activity was observed for extracts from *F.* × *intermedia* (up to 33%), with these extracts being more effective. Extracts from *G. globosa* and *C. ternatea* showed low inhibitory activity (<10%). The different activity between the extracts is explained by their different chemical composition [34,41,44,90]. The chemical composition of *P. officinalis* flowers is not well known, but our studies indicate the presence of significant amounts of gallic acid. This phenolic acid has the ability to inhibit AChE and has a relatively strong effect on BChE, as demonstrated in the studies of Orhan et al. [91]. Also, rutin and chlorogenic acid, present in extracts from *F.* × *intermedia*, show activity against both enzymes, but inhibit AChE more strongly than BChE [92]. Other compounds present in extracts from the remaining species did not significantly contribute to cholinesterase inhibition.

## 3. Materials and Methods

### 3.1. Chemical Reagents

Sodium carbonate, sodium hydroxide, DMSO, formic acid, methanol, ammonium acetate, and copper (II) chloride were purchased from Avantor Performance Materials Poland S.A. (Gliwice, Poland). The Folin–Ciocalteu phenol reagent and HPLC-grade acetonitrile were from Merck (Darmstadt, Germany). All other chemicals were from the Sigma–Aldrich Chemical Co. (Taufkirchen, Germany or St. Louis, MO, USA). High-quality pure water and ultra-high-quality pure water were prepared using a Direct-Q 3 UV Merck Millipore purification system (Merck, Darmstadt, Germany).

### 3.2. Plant Material

The plant material tested was the dried flowers of 4 different species: *P. officinalis*, *F.* × *intermedia*, *G. globosa*, and *C. ternatea*. The plant material from *F.* × *intermedia* came from the Botanical Garden of Adam Mickiewicz University in Poznan. The rest of the plant material was purchased from Nanga, Poland.

All plant samples are stored at the Department of Pharmacognosy and Biomaterials, Poznan University of Medical Sciences. The sample codes are as follows: PO_fl_1_2023, FI_fl_1_2023, CG_fl_1_2023, and CI_fl_1_2023. The *F.* × *intermedia* sample was identified by Dr. Mateusz Sowelo (Botanical Garden of Adam Mickiewicz University in Poznan). The samples of *G. globosa* and *C. ternatea* were commercially available and purchased.

### 3.3. Extraction Process

The experiments were performed on the flowers of four different plant species. The weighed portions (2.5 g) and the solvent (water, ethanol, or 50% ethanol) were placed in a glass Erlenmeyer flask sealed with a glass cork and parafilm. The extraction was performed in an ultrasonic bath, heated to 60 °C. Then, the extracts were filtered through cotton wool into a beaker, in which the filtrate from each repetition stage was combined. Due to the fourfold repetition of the extraction (20 min each), 200 mL samples were obtained. In the next stage, the volume of the extracts was decreased using a rotary evaporator. Lastly, extracts were supplemented with appropriate solvents, and their volume was equalised to 25 mL each.

### 3.4. Phytochemical Studies of the Extracts

#### 3.4.1. Total Polyphenol Content

The procedure was conducted following the method outlined by Studzińska-Sroka et al. with modifications [93]. Briefly, 25.0 µL of either the test extract or standard, followed by 200.0 µL of distilled water, 15.0 µL of the Folin–Ciocalteu reagent, and 60.0 µL of a 20% calcium carbonate solution, were successively added to the wells. A control sample containing only the reagents (excluding the test extract or standard) was included. The plate was then shaken in the dark for 30 min at 350 rpm at room temperature. Absorbance was recorded at 760 nm using a Multiskan GOx1510 microplate reader (Thermo-Scientific, Waltham, MA, USA). The experiment was carried out in duplicate. The results are the average of *n* = 6 measurements for the extract samples and *n* = 3 measurements for the reference. The results were reported as milligrams of gallic acid equivalents (GAE) per gram of dry plant material (DW), along with the standard deviation (SD).

#### 3.4.2. Total Flavonoid Content

The analysis was carried out according to the method described by Studzińska-Sroka et al. [93]. In short, 100.0 µL of the tested extract or reference compound was combined with 100.0 µL of a 2% methanolic aluminium chloride solution. After shaking the plate for 1 min, the mixture was incubated for 10 min in the dark at room temperature. Absorbance was measured at 415 nm using a Multiskan GO 1510 microplate reader (Thermo Fisher Scientific, Vantaa, Finland). The blank sample consisted of the extract mixed with methanol instead of the aluminium chloride solution. The experiment was carried out in duplicate. The results are the average of *n* = 6 measurements for the extract samples and of *n* = 3 measurements for the reference. The results are expressed as milligrams of quercetin equivalent (QE) per gram of dry plant material (DW), with standard deviation (SD).

#### 3.4.3. Standardisation of the Extract Using High-Performance Liquid Chromatography (HPLC)

The compounds selected for quantification were chosen based on their clear presence in HPLC profiles and their occurrence in the flowers of the studied species, as reported in the literature. Due to limited access to LC-MS, we focused on reliably detectable compounds with available standards. A high-performance liquid chromatography system (Dionex Thermoline Fisher Scientific, Dreieich, Germany) equipped with a high-pressure pump (UltiMate 3000), an autosampler (UltiMate 3000), and a DAD detector (UltiMate 3000) with Chromeleon software version 7.0 from Dionex Thermoline Fisher Scientific (Dreieich, Germany) was used to assess the presence and content of chlorogenic acid, ferulic acid, gallic acid, p-coumaric acid, and rutin in different flower extracts from *P. officinalis*, *F.* × *intermedia*, *G. globosa*, and *C. ternatea*. The analysis was performed according to the method described by Paczkowska-Walendowska et al. [94], and the procedure was validated to ensure the reliability of the results. The chromatographic conditions included a detection wavelength of 240 nm for chlorogenic acid and ferulic acid, 265 nm for gallic acid and p-coumaric acid, and 360 nm for rutin; a mobile phase flow rate of 1 mL/min; a column temperature of 40 °C; and a mobile phase consisting of 0.1% formic acid (solvent A) and acetonitrile (solvent B). The gradient elution was as follows: 0–35 min: 2–20% B; 35–55 min: 20–70% B; 55–60 min: 2% B. Standard solutions were prepared in HPLC-grade methanol at the following concentrations: gallic acid—0.1 and 0.01 mg/mL, ferulic acid—0.001, 0.01, and 0.1 mg/mL, p-coumaric acid—0.001, 0.01, and 0.1 mg/mL, and rutin—0.5 mg/mL. All solutions were filtered through a 0.45 µm membrane filter before injection. A series of injection volumes was applied for each compound and concentration to construct calibration curves. Injection volumes were as follows: for chlorogenic acid—4, 6, 8, and 10 µL (0.1 mg/mL), and 1, 2, 4, 6, 8, 10, and 12 µL (1.0 mg/mL); for gallic acid—2, 5, 10, 20, and 50 µL (0.01 mg/mL), and 10, 20, and 50 µL (0.1 mg/mL); for ferulic acid—5 µL (0.001 mg/mL), 1, 10, and 40 µL (0.01 mg/mL), 8, 16, and 32 µL (0.1 mg/mL); for p-coumaric acid—5 µL (0.001 mg/mL), 1, 10, and 40 µL (0.01 mg/mL), 8, 16, and 32 µL (0.1 mg/mL); rutin—5, 10, 20, 40, 60, and 80 µL (0.5 mg/mL). Calibration curves were established, and the method was validated by evaluating parameters such as linearity, direct and indirect precision, and the limits of detection (LOD) and quantification (LOQ). The results are expressed as milligrams of compound per 100 g of dry plant material (DW), with standard deviation (SD).

#### 3.4.4. GC-MS Analysis

Chromatographic studies were performed on a GC-MS chromatograph (SCION TQ, BRUKER). Approximately 1.15 ± 0.01 g of plant material (each flower tested) was finely ground using a mortar and pestle. Subsequently, 10 mL of chloroform (Thermo Scientific, spectroscopy grade) was added to the sample, and grinding was continued until a homogeneous solution was obtained. The resulting extract was then filtered through a 0.20 µm syringe filter. The filtrate was subjected to gas chromatography–mass spectrometry (GC-MS) analysis. For this step, 1.0 µL of the solution was injected onto the column. The chromatograph was equipped with a VF-5 ms Crawford Scientific silica column. The electron energy was 70 eV, and the ion source was 200 °C. Helium was used as the carrier gas at a 1.0 mL/min flow rate. Temperature programme: enable coolant at 50.0 °C, coolant timeout 20.00 min, stabilisation time 0.50 min; temperature 60.0 °C, hold 3.00 min, total 3.00 min; temperature 280.0 °C, rate 10.0 °C/min, hold 35.00 min, total 60.00 min. The compounds’ identification was based on comparing their retention time and mass spectra with NIST standards.

### 3.5. Studies of the Biological Activity of Extracts

#### 3.5.1. Antioxidant Activity Assay

##### DPPH Assay

A DPPH assay was employed to assess antiradical activity, following the procedure outlined in reference [95]. In brief, 25.0 µL of the test extract—prepared at a concentration of 2.5 mg of dry plant material per mL—was mixed with 175.0 µL of DPPH solution (3.9 mg in 50 mL of methanol). The mixture was shaken in the dark at 350 rpm for 5 min at room temperature and then incubated in the dark for an additional 25 min. Absorbance was recorded at 517 nm using a microplate reader (Multiskan GO 1510, Thermo Fisher Scientific, Vantaa, Finland). All analyses were performed in duplicate, and the final results represent the mean of five measurements (*n* = 5). Results are expressed as % ± standard deviation (SD).

##### ABTS Assay

An ABTS assay was also employed to assess antiradical activity. It followed the procedure outlined in reference [96]. In short, 10.0 µL of the test extract (2.5 mg of dry plant material per mL) was mixed with 200.0 µL of ABTS solution (0.1920 g ABTS-(NH4)_2_ in 50 mL of 0.3311 mg K_2_S_2_O_8_ in 500 mL of distilled water). Before use, the basic ABTS solution was diluted so that the absorbance was ~0.77 with a 734 nm wavelength. The mixture was shaken in the dark at 500 rpm for 30 min at room temperature. Absorbance was recorded at 734 nm using a microplate reader (Multiskan GO 1510, Thermo Fisher Scientific, Vantaa, Finland). All analyses were performed in duplicate, and the final results represent the mean of five measurements (*n* = 5). Results are expressed as % ± standard deviation (SD).

##### CUPRAC Assay

A CUPRAC assay was performed following a previously described method [97]. The CUPRAC reagent was prepared by combining equal volumes of neocuproine (7.5 mM), copper (II) chloride (10 mM), and ammonium acetate buffer (1 M, pH 7.0). Subsequently, 50 µL of the test extract (2.5 mg dry plant material/mL) was mixed with 150 µL of the reagent. After incubating the mixture in the dark with shaking (350 rpm) for 30 min at room temperature, absorbance was measured at 450 nm. A blank sample was prepared by substituting the extract with an equal volume of the extraction solvent. The experiment was conducted in duplicate, and results are expressed as the mean of four measurements (*n* = 4). The test results were expressed as mg of Trolox per gram of dry weight ± standard deviation (SD).

##### FRAP Assay

A FRAP assay was carried out based on the method described by Tiveron et al. [98] with slight modifications. The stock FRAP reagent was composed of a 300 mM acetate buffer (pH 3.6), a 10 mM TPTZ solution prepared in 40 mM HCl, and a 20 mM FeCl_3_·6H_2_O solution. The working FRAP reagent was freshly made by combining 25 mL of acetate buffer, 2.5 mL of the TPTZ solution, and 2.5 mL of the FeCl_3_·6H_2_O solution, and then preheated to 37 °C before use. For the assay, 20 µL of the test extract (2.5 mg dry plant material/mL) was mixed with 180 µL of the FRAP reagent and incubated at 37 °C for 30 min in the dark. For the blank FRAP reagent, TPTZ solution was used instead. Absorbance was measured at 593 nm in four replicates (*n* = 4). The test results were expressed as mg of Trolox per gram of dry weight. The test results were expressed as mg of Trolox per gram of dry weight ± standard deviation (SD).

##### Chelation Power on Ferrous (Fe^2+^) Ions Assay

The Fe^2+^ chelating ability of the extracts was assessed based on the method by Dinis et al. [99], with modifications. In summary, 200.0 μL of the sample solution at a concentration of 2.5 mg of dry plant material per mL was mixed with 10.0 μL of FeCl_2_·4H_2_O (1 mM) and pre-incubated in a 96-well plate at room temperature for 10 min (with shaking, 500 rpm). Then, 40.0 μL of ferrozine (2.5 mM) was added, and the mixture was incubated for 30 min at room temperature (with shaking, 500 rpm). The absorbance of the Fe^2+^–ferrozine complex was recorded at 562 nm in five replicates (*n* = 5). The chelating activity, corresponding to the final sample concentration, was calculated and expressed as % of chelating activity ± standard deviation (SD).

#### 3.5.2. Inhibition of Carbohydrate-Hydrolysing Enzymes

##### α-Glucosidase Inhibition Assay

The α-glucosidase inhibitory activity of the extracts was assessed following the method of Studzińska-Sroka et al. [93], with slight modifications. In short, 50.0 µL of the extract (25.0 mg dry plant material/mL) was pre-incubated with 50.0 µL of 0.1 M phosphate buffer (pH 6.8) and 30.0 µL of α-glucosidase solution (0.5 U/mL) in a 96-well plate at 37 °C for 15 min. Afterwards, 20.0 µL of a 5 mM p-nitrophenyl-α-D-glucopyranoside (pNPG) solution in 0.1 M phosphate buffer was added, and the mixture was incubated for 20 min at 37 °C. The reaction was terminated by adding 100.0 µL of 0.2 M sodium carbonate. Absorbance was read at 405 nm using a Multiskan GO 1510 microplate reader (Thermo Fisher Scientific, Vantaa, Finland), 2 min after stopping the reaction. Control measurements were performed without extract, while blanks consisted of extract or standard solutions without enzyme. Results represent the mean of three replicates (*n* = 3) and are expressed as % of inhibition ± standard deviation (SD).

##### α-Amylase Inhibition Assay

The α-amylase inhibitory activity of the extracts was evaluated following the protocol described by Studzińska-Sroka et al. [93], with slight modifications. Briefly, 50.0 µL of the extract (25.0 mg dry plant material/mL) was preincubated with 20 µL of α-amylase solution (2.0 U/mL, prepared in phosphate buffer at pH 6.9) at 37 °C for 20 min. Subsequently, 20.0 µL of a 0.5% starch solution in phosphate buffer was added, and the mixture was incubated again at 37 °C for another 20 min. Then, 60 µL of a colour reagent (containing 96 mM 3,5-dinitrosalicylic acid, 5.31 M potassium sodium tartrate in 2 M sodium hydroxide, and deionised water) was added, and the plate was heated at 90 °C. After 45 min, 100 µL of distilled water was added, and the absorbance was measured at 540 nm using a microplate reader (Multiskan GO 1510, Thermo Fisher Scientific, Vantaa, Finland). The control sample included 20 µL of distilled water with 20 µL of amylase solution, while the blank consisted of 20 µL of distilled water and 20 µL of phosphate buffer. Results represent the average of four replicates (*n* = 4), and are expressed as % of inhibition ± standard deviation (SD).

#### 3.5.3. Anti-Inflammatory Activity Assay

##### Hyaluronidase Inhibition Assay

The anti-hyaluronidase activity assay followed a previously reported procedure [100], with adjustments. In short, 25.0 µL of incubation buffer, 25.0 µL of hyaluronidase enzyme solution (30 U/mL), 10.0 µL of the test extract (25 mg of dry plant material per mL), and 15.0 µL of acetate buffer were combined in a well. The mixture was incubated at 37 °C for 15 min with shaking at 200 rpm. Subsequently, 25.0 µL of hyaluronic acid (HA) solution was added, followed by a second incubation for 45 min under the same conditions. Afterwards, 200.0 µL of cetyltrimethylammonium bromide (CTAB) solution in 2% NaOH was introduced, and the mixture was incubated at room temperature for 10 min without shaking. Absorbance was measured at 600 nm using a microplate reader (Multiskan GO 1510, Thermo Fisher Scientific, Vantaa, Finland). The blank composition was as previously described [101]. The assay was performed in duplicate, and values were calculated from four measurements (*n* = 4). Results are expressed as % of inhibition ± standard deviation (SD).

##### Determination of Anti-Inflammatory Potential in RAW 264.7 Model

Murine RAW 264.7 macrophages were cultured at 37 °C, in a 5% CO_2_ atmosphere, with relative humidity, using DMEM high glucose, supplemented with 10% foetal bovine serum as the culture medium. Before the anti-inflammatory experiment, the toxic potential of the tested samples to the macrophages was evaluated using MTT assay, in the concentration range of 10–200 μg/mL, as described previously [97]. For the determination of anti-inflammatory potential, the macrophages were seeded onto 96-well plates (1.5 × 10^5^ cells/well) and pre-treated with the tested samples (at the concentration 10–200 μg/mL) or dexamethasone (0.5 μg/mL) as a reference drug, for 1 h. Next, inflammation was induced by adding 10 ng/mL of LPS to each well, as previously described [97], and the incubation was performed for 24 h. Cells treated with LPS alone were used as a positive control, while cells not treated with either tested samples or LPS were included as the untreated control. The values were calculated from three measurements (*n* = 3). Nitric oxide level was determined in cell culture supernatants with Griess Reagent Kit (Promega Corporation, Madison, Winooski, VT, USA), according to the manufacturer’s protocol. The results were expressed as a % of the LPS control.

#### 3.5.4. Anti-Cholinesterase Activity Assay

##### AChE and BChE Inhibition Activity Assays

The studied extracts were dissolved in an appropriate solvent to a concentration of 25 mg/mL. The cholinesterase inhibitory activity was assessed using Ellman’s spectrophotometric method [102], incorporating modifications previously described by Studzińska-Sroka et al. [103]. In brief, 5.0 µL of each test sample was combined with 60.0 µL of TRIS-HCl buffer (50 mM, pH 8.0) and 30.0 µL of AChE or BChE solution (0.2 U/mL). The mixture was incubated for 5 min at 25 °C with shaking at 500 rpm. Following this, 30.0 µL of acetylthiocholine iodide (1.5 mM) and 125.0 µL of 5,5′-dithiobis(2-nitrobenzoic acid) solution (0.3 mM, containing 10 mM NaCl and 2 mM MgCl_2_·6H_2_O) were added, and incubation continued for 30 min under the same conditions. Sample blanks were prepared by replacing the enzyme with buffer. The control sample included an appropriate solvent instead of the test substance, and its blank replaced both the enzyme and the sample with buffer and an appropriate solvent, respectively. Absorbance was recorded at 405 nm using a Multiskan GO 1510 microplate reader (Thermo Fisher Scientific, Vantaa, Finland). Each extract was tested in two independent experiments, and the results represent the mean of five measurements (*n* = 5).

### 3.6. Statistical Analysis

Statistical calculations were performed using Microsoft Excel (Microsoft Office LTSC 2021 Professional Plus, Microsoft Corp., Redmond, WA, USA). Data are presented as mean ± standard deviation (SD). One-way analysis of variance (ANOVA) was used to evaluate differences between groups. Post hoc comparisons were performed using the Newman–Keuls test to assess differences between all experimental groups and the Dunnett test to compare each treatment group with the LPS-treated control. Statistical significance was considered at *p* < 0.05.

## 4. Conclusions

The present study shows that edible flower extracts—especially those from *P. officinalis* and *F.* × *intermedia*—possess interesting biological potential that may contribute to the prevention or adjuvant treatment of type 2 diabetes and its complications. Among the species studied, *P. officinalis* extracts, especially those obtained with 50% ethanol, showed the highest total polyphenol content and strong antioxidant activity in many tests performed (DPPH, ABTS, CUPRAC, and FRAP), together with the strong inhibition of α-glucosidase and α-amylase, suggesting a broad spectrum of bioactivity. High levels of gallic acid and rutin in this species probably contribute to these effects. Similarly, *F.* × *intermedia* extracts showed high rutin and chlorogenic acid contents and selective enzyme inhibition, with aqueous extracts showing the potent inhibition of α-amylase and hyaluronidase. This result further emphasised that the type of extract may be crucial for the direction of action shown. Although *C. ternatea* extracts showed a moderate polyphenol content, the ethanol–water extract of the raw material showed a noticeable inhibition of NO release in macrophage assays, indicating promising anti-inflammatory properties. In contrast, *G. globosa* extracts generally showed a low polyphenol content and weak antioxidant and anti-inflammatory activity, although ethanol extracts showed significant α-amylase inhibition. In conclusion, this study highlighted the value of edible flowers—especially *P. officinalis* and *F.* × *intermedia*—as a rich source of bioactive phytochemicals with antioxidant, antidiabetic, and anti-inflammatory properties. These findings may be useful for further studies on their therapeutic potential for alleviating oxidative stress, hyperglycemia, and chronic inflammation associated with metabolic disorders.

## Figures and Tables

**Table 1:** The selected phenolic acids and flavonoid content in the extracts of four edible flower species (*Peonia officinalis*, *Forsythia* × *intermedia*, *Gomphrena globosa*, *Clitoria ternatea*) determined using HPLC.

Extract	Compound Detected Amount [mg/100 g DW]				
	Gallic Acid	Chlorogenic Acid	p-Coumaric Acid	Ferulic Acid	Rutin
P-100% EtOH	493.31 ± 18.43 ^b^	nd	nd	nd	239.59 ± 29.66 ^c^
P-50% EtOH	390.27 ± 15.48 ^c^	nd	nd	nd	167.65 ± 0.56 ^d^
P-H_2_O	603.67 ± 10.10 ^a^	nd	nd	nd	207.56 ± 0.41 ^c,d^
F-100% EtOH	1.42 ± 0.07 ^d^	319.79 ± 4.21 ^b^	nd	6.37 ± 0.64 ^e^	2828.71 ± 41.11 ^b^
F-50% EtOH	19.87 ± 3.36 ^d^	796.03 ± 2.4 ^a^	1.11 ± 0.09 ^c,d^	8.80 ± 1.12 ^d^	4657.71 ± 66.37 ^a^
F-H_2_O	4.82 ± 0.54 ^d^	0.31 ± 0.28 ^c^	1.11 ± 0.38 ^c,d^	0.33 ± 0.30 ^h^	0.90 ± 0.31 ^f^
G-100% EtOH	0.08 ± 0.06 ^d^	2.83 ± 0.37 ^c^	0.88 ± 0.06 ^d^	4.53 ± 1.18 ^f^	34.90 ± 0.38 ^f^
G-50% EtOH	0.05 ± 0.07 ^d^	0.70 ± 0.47 ^c^	0.86 ± 0.09 ^d^	6.05 ± 1.10	38.44 ± 0.37 ^f^
G-H_2_O	0.17 ± 0.10 ^d^	2.55 ± 0.19 ^c^	5.07 ± 0.10 ^a^	12.18 ± 0.23 ^c^	23.39 ± 0.27 ^f^
C-100% EtOH	nd	0.17 ± 0.04 ^c^	0.06 ± 0.02 ^e^	3.17 ± 0.01 ^g^	11.69 ± 0.89 ^f^
C-50% EtOH	nd	1.93 ± 0.83 ^c^	1.32 ± 0.08 ^c^	85.95 ± 1.02 ^a^	160.35 ± 0.99 ^d^
C-H_2_O	nd	1.59 ± 0.16 ^c^	1.70 ± 0.35 ^b^	57.76 ± 0.69 ^b^	91.87 ± 4.47 ^e^

nd—not detected. Values are presented as mean ± SD. Different letters indicate significant differences between groups (*p* < 0.05, one-way ANOVA followed by Newman–Keuls post hoc test).

**Table 2:** Compounds identified in the extracts of four edible flower species (*Peonia officinalis*, *Forsythia* × *intermedia*, *Gomphrena globosa*, *Clitoria ternatea*) using GC-MS method.

Plant	Rt (min)	Quantity (%)	Compound Name	Formula	Main Characteristic *m*/*z*
*Peonia officinalis*	15.75	1.53	Propanoic acid, 2-(3-acetoxy-4,4,14-trimethylandrost-8-en-17-yl)-	C_27_H_42_O_4_	73, 147, 207, 281, 355, 444
	22.42	8.83	Octadecane, 3-ethyl-5-(2-ethylbutyl)-	C_26_H_54_	57, 71, 85, 99, 141, 207
	23.236	7.62	Oleic acid, 3-(octadecyloxy)propyl ester	C_39_H_76_O_3_	57, 207, 281, 407, 462, 519
	24.022	47.31	Pentacosane	C_25_H_52_	57, 71, 85, 99, 127, 141, 207, 225, 281, 322
	25.552	29.15	Tetratetracontane	C_44_H_90_	57, 71, 85, 97, 207, 281, 326, 372., 423, 533
	26.191	5.56	Spirost-8-en-11-one, 3-hydroxy-, (3ß,5a,14ß,20ß,22ß,25R)-	C_27_H_40_O_4_	57, 69, 96, 207, 281, 355, 415
*Forsythia* × *intermedia*	22.188	1.462	2-Hexadecanol	C_16_H_34_O	55, 69, 97, 207
	22.292	0.349	Oleic Acid	C_18_H_34_O_2_	55, 69, 83, 97, 207, 237, 281
	22.405	3.931	Hexadecane, 2,6,10,14-tetramethyl-	C_20_H_42_	57, 71, 85, 99, 113, 141, 207, 238, 266
	23.069	1.209	9-Octadecenamide	C_18_H_35_NO	55, 59, 72, 83, 154, 207, 281
	23.724	2.102	Heptacosane	C_27_H_56_	57, 71, 85, 99, 127, 141, 183, 207, 239, 388
	23.824	1.177	17-Pentatriacontene	C_35_H_70_	57, 69, 83, 97, 207, 281, 350, 379, 445
	24.017	3.915	Hentriacontane	C_31_H_64_	57, 71, 85, 99, 127, 155, 183, 207, 239, 265, 295, 323, 352
	25.544	4.212	Tetratetracontane	C_44_H_90_	57, 71, 85, 97, 133, 207, 281, 337, 383, 575, 624
	27.07	0.370	Ergosta-5,22-dien-3-ol, acetate, (3ß,22E)-	C_30_H_48_O_2_	57, 67, 91, 133, 145, 207, 281, 341, 393
	27.486	1.602	Octadecane, 3-ethyl-5-(2-ethylbutyl)-	C_26_H_54_	57, 71, 85, 99, 127, 207, 281
	30.858	79.670	Dihydrofuran-2-one, 4-(3,4-dimethoxybenzyl)-3-(4-hydroxy-3-methoxybenzyl)-	C_21_H_24_O_6_	77, 122, 137, 151, 177, 189, 207, 235, 265, 293, 321, 372
*Gomphrena globosa*	21.838	7.208	3,10B-Dihydrofluoranthene	C_16_H_12_	101, 202, 282
	23.114	3.658	5,7,9(11)-Androstatriene, 3-hydroxy-17-oxo-	C_19_H_24_O_2_	59, 77, 115, 133, 195, 207, 284
	24.03	1.075	Oleic acid, 3-(octadecyloxy)propyl ester	C_39_H_76_O_3_	57, 133, 207, 281, 309, 403
	25.551	4.978	Heptacosane	C_27_H_56_	57, 71, 85, 99, 113, 207, 281, 341, 389
	27.492	41.740	Tetratetracontane	C_44_H_90_	57, 71, 85, 97, 127, 207, 281, 377, 429
	30.315	30.131	Hexatriacontane	C_36_H_74_	57, 71, 85, 99, 127, 155, 207, 281, 322, 391, 436
	34.609	11.210	Tetracosane, 11-decyl-	C_34_H_70_	57, 71, 85, 99, 207, 281, 355, 402
*Clitoria ternatea*	11.612	0.917	Spironolactone	C_24_H_32_O_4_S	55, 73, 91, 147, 207, 251, 341, 429
	13.807	1.117	Octadecane, 1,1′-[1,3-propanediylbis(oxy)]bis-	C_39_H_80_O_2_	55, 73, 85, 97, 147, 207, 251, 281, 327, 415, 504
	18.137	4.952	17-Octadecynoic acid	C_18_H_32_O_2_	55, 68, 81, 95, 123, 207, 281
	18.254	2.652	2-Hexadecanol	C_16_H_34_O	55, 69, 79, 95, 123, 207, 237
	18.389	2.459	Ethanol, 2-(9-octadecenyloxy)-, (Z)-	C_20_H_40_O_2_	55, 69, 81, 96, 207, 250, 281, 325
	18.585	3.424	13-Heptadecyn-1-ol	C_17_H_32_O	55, 67, 81, 95, 123, 207, 278
	25.549	4.120	Heptacosane	C_27_H_56_	57, 71, 85, 99, 133, 207, 281, 355, 402
	27.489	14.287	Tetratetracontane	C_44_H_90_	57, 71, 85, 97, 127, 207, 281, 306, 347, 391, 439, 481, 535
	30.311	49.099	Octadecane, 3-ethyl-5-(2-ethylbutyl)-	C_26_H_54_	57, 71, 85, 97, 127, 155, 207, 281, 322, 355
	34.602	16.309	Hexatriacontane	C_36_H_74_	57, 71, 85, 95, 207, 281, 341
	36.634	0.662	Benz[e]azulene-3,8-dione, 3a,4,6a 7,9,10,10a,10b-octahydro-3a,10a-dihydroxy-5-(hydroxymethyl)-7-(1-hydroxy-1-methylethyl)-2,10-dimethyl-, [3aR-(3aa,6aa,7a,10ß,10aß,10bß)]-	C_20_H_28_O_6_	53, 69, 73, 69, 133, 179, 207, 281, 314

**Table 3:** Antioxidant activity assays—DPPH, ABTS, Fe^2+^ chelation, CUPRAC, and FRAP.

	DPPH [%]	ABTS [%]	Fe^2+^ chelation [%]	CUPRAC [mg TE/g DW]	FRAP [mg TE/g DW]
P-100% EtOH	97.65 ± 0.60 ^a^	100.00 ± 0.99 ^a^	nd	175.06 ± 1.04 ^a^	144.06 ± 4.56 ^a^
P-50% EtOH	90.42 ± 0.89 ^c^	99.51 ± 0.41 ^a^	73.78 ± 2.58 ^c^	151.44 ± 2.18 ^c^	140.29 ± 1.10 ^b^
P-H_2_O	91.01 ± 0.41 ^c^	99.78 ± 0.18 ^a^	83.36 ± 0.44 ^a^	162.04 ± 0.01 ^b^	137.52 ± 3.09 ^c^
F-100% EtOH	95.48 ± 0.27 ^b^	65.09 ± 1.02 ^b^	nd	72.58 ± 0.87 ^e^	51.94 ± 0.21 ^e^
F-50% EtOH	89.28 ± 0.64 ^d^	99.10 ± 0.53 ^a^	52.76 ± 1.32 ^e^	109.30 ± 2.82 ^d^	81.30 ± 0.76 ^d^
F-H_2_O	28.44 ± 0.49 ^e^	43.26 ± 0.91 ^e^	77.38 ± 0.56 ^b^	24.24 ± 0.49 ^f^	20.36 ± 0.33 ^f^
G-100% EtOH	10.56 ± 0.93 ^i^	10.50 ± 0.32 ^i^	nd	6.32 ± 0.05 ^i^	5.36 ± 0.02 ^f^
G-50% EtOH	6.89 ± 0.84 ^j^	15.82 ± 0.90 ^h^	52.68 ± 4.27 ^e^	7.81 ± 0.31 ^h,i^	7.36 ± 0.07 ^g^
G-H_2_O	12.34 ± 0.37 ^h^	29.63 ± 0.94 ^f^	80.81 ± 0.90 ^a^	8.56 ± 0.49 ^h^	8.37 ± 0.04 ^g^
C-100% EtOH	15.87 ± 1.78 ^g^	20.30 ± 0.76 ^g^	nd	8.90 ± 0.35 ^h^	5.83 ± 0.04 ^g^
C-50% EtOH	21.83 ± 0.77 ^f^	54.36 ± 1.60 ^c^	58.62 ± 6.27 ^d^	23.41 ± 0.40 ^f^	20.30 ± 0.24 ^f^
C-H_2_O	22.06 ± 0.72 ^f^	47.43 ± 1.71 ^d^	71.28 ± 3.24 ^c^	21.70 ± 0.12 ^g^	19.46 ± 0.17 ^f^

P—*Paeonia officinalis*, F—*Forsythia* × *intermedia*, G—*Gomphrena globosa*, C—*Clitoria ternatea*. Values are presented as mean ± SD. Different letters indicate significant differences between groups (*p* < 0.05, one-way ANOVA followed by Newman–Keuls post hoc test).

## Data Availability

All data supporting reported results can be found within the manuscript.
